# Accelerated atherosclerosis in rheumatoid arthritis: a systematic review

**DOI:** 10.12688/f1000research.112921.1

**Published:** 2022-04-27

**Authors:** Rhea Raj, Sneha Thomas, Vasavi Gorantla

**Affiliations:** 1 St. George's University School of medicine, True Blue, St. George's, Grenada; 2 University of Maryland Medical Center MTC, Midtown, Baltimore, USA

**Keywords:** Rheumatoid arthritis, atherosclerosis, atherogenesis, premature, pathophysiology, inflammation, cytokines, endothelial dysfunction, autoantibodies

## Abstract

**Background:** Rheumatoid arthritis (RA) is a highly prevalent, chronic inflammatory condition of the synovial joints that affects approximately 1% of the global population. The pathogenesis of RA is predominantly inflammatory in nature, thereby accelerating the co-occurrence of other immunoinflammatory conditions such as atherosclerosis. Apart from traditional cardiovascular risk factors, RA patients possess a multitude of other factors that predispose them to early atherosclerotic disease. The aim of this systematic review is to assess the prevalence of premature atherosclerosis in RA patients and elucidate the role that proinflammatory cytokines, neutrophil extracellular traps, RA-related autoantibodies, and endothelial dysfunction play in the pathophysiology of RA-mediated atherosclerosis. We also discussed novel biomarkers that can be used to predict early atherosclerosis in RA.

**Methods: **This review followed the PRISMA guidelines to select and analyze relevant articles. A literature search for articles was performed on February 25, 2022, through three research databases including PubMed, ProQuest, and ScienceDirect. The query used to identify relevant publications was “Rheumatoid arthritis and atherosclerosis” and the search duration was set from 2011-2022. Relevant articles were selected based on the inclusion and exclusion criteria.

**Results: **Our initial search generated 21,235 articles. We narrowed our search according to the inclusion and exclusion criteria. After assessing eligibility based on the full content of the articles, 73 articles were ultimately chosen for this review.

**Conclusion: **There is a high prevalence of accelerated atherosclerosis among RA patients. We found evidence to explain the role of proinflammatory cytokines, neutrophil extracellular traps, RA-related autoantibodies, and endothelial dysfunction in the pathophysiology RA-mediated atherosclerosis. Therapies targeting either the inflammatory load or traditional CV risk-factors seem to improve vascular outcomes in RA patients. Novel markers of atherosclerosis in RA may be useful in predicting premature atherosclerosis and serve as new targets for therapeutic intervention.

## Introduction and background

Rheumatoid arthritis (RA) is an autoimmune disorder, often described as a debilitating condition, that severely impairs quality of life by causing extra-articular manifestations. ^1^^–^^3^ RA affects approximately 1% of the global population and poses a significant societal and economic burden in terms of cost and disability. ^4^^,^^5^ The incidence of comorbidities such as atherosclerosis-related cardiovascular diseases, lung cancer, osteoporosis, and depression are higher among individuals with RA, making it a multisystem disease. ^6^ RA is described as a chronically progressive inflammatory condition that affects the synovial lining of joints in the fingers, wrists, feet, and ankles. ^1^ Since the pathological mechanism that lead to RA is predominantly inflammatory in nature, it facilitates the co-occurrence of other immunoinflammatory conditions such as atherosclerosis. ^7^^,^^8^ Atherosclerosis refers to the hardening of an artery due to the buildup of fatty, cholesterol-rich plaque within the intimal lining of the vessel wall. ^8^^,^^9^ The pathophysiological link between RA and atherosclerosis has its roots in complex inflammatory pathways that interconnect the two conditions and serve as an explanation for the increased cardiovascular morbidity in RA patients. ^10^^,^^11^ Tumor necrosis factor alpha (TNF-α), is a proinflammatory cytokine that is highly elevated in the synovial fluid of individuals with RA. ^12^^,^^13^ TNF-α, along with interleukin-6 (IL-6), promotes the accumulation of oxidized low density lipoprotein (oxLDL) within the vessel wall, which directly contributes to the formation of lipid-laden macrophages, also known as foam cells. ^13^^–^^15^ Macrophagic foam cells are considered to be the prototypical cells involved in the development of atherosclerotic plaques. ^15^^,^^16^ Interleukin-1 (IL-1) is another cytokine associated with RA that shares its proinflammatory properties with TNF-α as they both upregulate the expression of adhesion molecules on vascular endothelial surfaces, stimulate cytokine production, and induce the expression of proinflammatory genes, all of which favor the initiation of atherogenesis. ^17^^–^^20^

The role of neutrophil extracellular traps (NETs) in the pathogenesis of RA has been increasingly gaining attention. ^21^ NETs are a complex network of granular proteins, nuclear chromatin, and extracellular fibers that eliminate pathogens through the activation of the ROS-mediated suicidal NETosis pathway. ^21^^–^^23^ The role of neutrophils in atherogenesis has been historically denied, but recent evidence shows that neutrophils are involved in progressive endothelial damage, recruitment of proinflammatory monocytes, and foam cell formation, thus implicating them in the process of atherosclerosis. ^24^^–^^26^ The presence of citrullinated proteins within the synovia of patients with RA has been recognized as a target for anti-citrullinated peptide antibodies (ACPAs). ^27^^,^^28^ Citrullinated fibrinogen within atherosclerotic plaques have also shown to be targeted by RA-derived ACPAs, contributing to the development of atherosclerosis in the setting of RA. ^27^^,^^29^ It is well known that atherosclerosis is a consequence of progressive endothelial damage and dysfunction. ^30^

There is ample evidence to support the presence of both micro- and macrovascular endothelial dysfunction in RA which helps to strengthen the pathophysiological link between the two entities. ^31^ Our systematic review will assess the prevalence of premature atherosclerosis in RA patients and elucidate the role that proinflammatory cytokines, neutrophil extracellular traps, RA-related autoantibodies, and endothelial dysfunction play in the pathophysiology of RA-mediated atherosclerosis through analysis of available literature. We will also discuss carotid intima media thickness, flow mediated dilation, lipoprotein-associated phospholipase A2 enzyme activity, osteocalcin and osteoprotegerin levels as markers of predicting early atherosclerosis in RA patients.

## Methods

**Figure 1.**
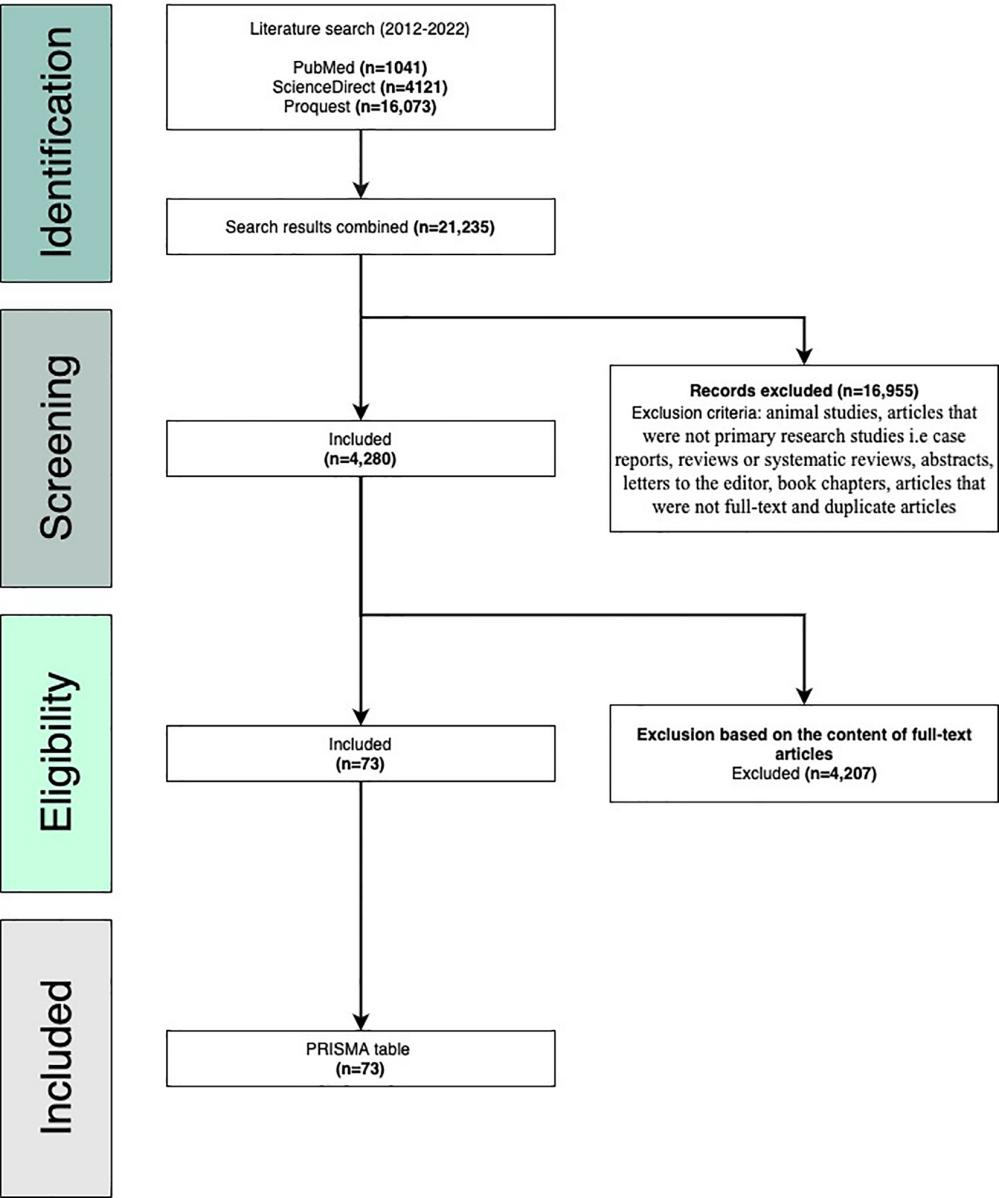
Preferred Reporting Items for Systematic Reviews (PRISMA) flow diagram.

This review strictly follows the Preferred Reporting Items for Systematic Reviews and Meta-analyses (PRISMA) guidelines as this is a widely accepted and validated methodology for choosing relevant publications to be included in systematic reviews. ^32^ A literature search for articles was performed on February 25, 2022, through three research databases: PubMed, ProQuest, and ScienceDirect. The query used to identify relevant publications was “Rheumatoid arthritis and atherosclerosis” and the search duration was set from 2011–2022. We included primary case–control studies, cohort studies, cross-sectional studies, observational studies, comparative studies, and meta-analyses. Once the search was complete, three co-authors worked independently to screen the results and extract data from each article. We acknowledge that despite our maximum efforts, some relevant articles may have been left off accidently. Our initial search generated 21,235 articles. Using manual screening, we narrowed the search according to the inclusion and exclusion criteria and a total of 73 articles were ultimately included in this systematic review ( Figure 1).

### Inclusion criteria

The following inclusion criteria were used: research studies conducted on humans and written in English, studies published in or after 2011, studies relevant to our topic of interest (accelerated atherosclerosis in rheumatoid arthritis), and articles that were full text, peer-reviewed, and primary or original research publications.

### Exclusion criteria

The following criteria were used for exclusion: animal studies, articles that were not primary research studies *i.e.* case reports, reviews or systematic reviews, abstracts, letters to the editor, book chapters, articles published outside of range (2011–2022), and articles not relevant to our review. All duplicates and non-full-text articles were also excluded. This information is visually presented in the PRISMA flow diagram ( Figure 1).

**Data items**

The information collected from each study included the name of the first author, year of publication, study design, study population, study aim, findings, and conclusion.

**Risk of bias in selected studies**

Once studies were selected, three co-authors were to grade the risk of bias in individual studies using using the GRADE (Grading of Recommendations Assessment, Development, and Evaluation) system. GRADE evaluates study flaws such as bias risk, indirectness, imprecision, and publication bias. Two reviewers would have used these criteria on a study-by-study basis, with a third reviewer assessing and providing an outcome if there was any inconsistency.

## Results

Our literature search yielded 21,235 articles: 1,041 from PubMed, 4121 from ScienceDirect, and 16,073 from ProQuest. 16,955 articles were excluded based on the exclusion criteria (animal studies, articles that were not primary research studies *i.e.* case reports, reviews or systematic reviews, abstracts, letters to the editor, book chapters, articles published outside of range, and articles not relevant to our review). All duplicates and non-full-text articles were also excluded. This resulted in 4,280 articles to be checked for eligibility. 73 articles were relevant and included in this review. Ultimately, there were 37 prospective studies, 10 case-control studies, eight cross-sectional studies, five comparative studies, three observational studies, three prospective observational studies, three meta-analyses, three retrospective studies, and one cohort analysis included in this review. The study characteristics are tabulated in Table 1.

**Table 1.** Baseline characteristics of the included studies.

	Author	Country	Design & study population	Aim	Findings	Conclusion
1	Ruscitti *et al.*, 2017 ^33^	Italy	Prospective observational study (n=347)	To investigate the existence of atherosclerosis in RA.	There was a significant increase in the development of subclinical atherosclerosis in RA patients during the follow-up (p<0.00001).	Both chronic inflammation and traditional cardiovascular risk factors are responsible for the occurrence of premature atherosclerosis in RA.
2	Ambrosino *et al.*, 2015 ^34^	Italy	Meta-analysis (n=7923)	To assess the impact of RA on cIMT and carotid plaques.	RA patients had a higher common carotid artery intima media thickness (CCA-IMT) (p<0.00001) and a higher prevalence of plaques (p<0.00001) compared with the control group.	RA is associated with subclinical atherosclerosis and regular CVD screening and prevention strategies would benefit RA patients.
3	Bes *et al.*, 2018 ^35^	Turkey	Case–control study (n=65)	To evaluate whether Lp-PLA2 activity was associated with atherosclerosis in RA.	Lipoprotein-associated phospholipase A2 (Lp-PLA2) enzyme activity was found to be similar in RA patients and healthy control subjects but lower than in patients with diabetes mellitus (DM) (p=0.006).	There was no association between Lp-PLA2 atherogenic activity and RA when compared to the control group and DM group, possibly because the RA patients were undergoing therapy and had low disease activity scores.
4	Ruscitti *et al.*, 2019 ^36^	Italy	Prospective observational study (n=841)	To determine occurrence of atherosclerosis in RA.	RA patients had a significantly increased rate of subclinical and clinical atherosclerosis (p<0.0001).	The long-term vascular outcomes of RA patients can be improved by recognizing the role of inflammation and traditional CVD risk factors in order to develop treatment regimens accordingly.
5	Verma *et al.*, 2017 ^37^	India	Prospective study (n=60)	To assess endothelial function in patients with RA.	In RA patients, flow mediated dilatation (FMD) was found to be significantly lower than the control group (p<0.001) whereas cIMT was found to be higher in RA patients compared with controls (p=0.003).	These findings were consistent with endothelial dysfunction and accelerated atherosclerosis in patients with RA.
6	Hannawi *et al.*, 2020 ^38^	UAE	Cross-sectional study (n=250)	To assess the occurrence of subclinical atherosclerosis in RA.	Patients with RA had a significantly higher cIMT compared with the control group (p=0.03).	This study concluded that subclinical atherosclerosis is more prevalent in RA patients compared with healthy controls.
7	Krajnc *et al.*, 2021 ^39^	Slovenia	Prospective observational study (n=110)	To assess the importance of traditional CV factors and inflammation in RA.	Both plaque formation (p=0.005) and cIMT (p=0.001) was greater in patients with RA compared with the matched control group.	Inflammatory mediators seem to be more significant than traditional CVD risk factors as indicators of the atherosclerotic burden in premenopausal women with RA.
8	Burggraaf *et al.*, 2018 ^40^	The Netherlands	Cohort analysis (n=212)	To assess cIMT in RA patients following a treat-to-target intervention and in patients with concomitant MetS.	Treat-to-target intervention resulted in lower progression of cIMT. In RA patients with metabolic syndrome (MetS), carotid intima media thickness (cIMT) was higher than in patients without MetS (p<0.001).	In RA patients without MetS, implementing CVD therapeutic regimens can help curb the progression of atherosclerotic plaques.
9	Suarez *et al.*, 2019 ^41^	Mexico	Case–control study (n=209)	To evaluate carotid ultrasound findings in RA patients.	The incidence of bilateral carotid plaque (CP) was more than double in RA patients compared with the control group (15.5% *versus* 6.6%).	This study confirmed the increased prevalence of RA-related subclinical atherosclerosis.
10	Ursini *et al.*, 2017 ^42^	Italy	Meta-analysis (n=346)	To assess whether anti-TNF-α therapy improves endothelial function.	RA patients receiving anti-TNF-α therapy showed a statistically significant improvement of endothelial function (p<0.0001).	Endothelial dysfunction is one of the mechanisms implicated in the increased cardiovascular morbidity in individuals with RA.
11	Végh *et al.*, 2019 ^43^	Hungary	Prospective cohort study (n=53)	To assess whether anti-TNF-α therapy improves vascular function in RA patients.	Anti-TNFα treatment showed an improvement in FMD and pulse wave velocity (PWV) in patients with RA during the course of the study (p=0.004 and (p=0.034 respectively).	This shows that TNFα inhibition improved and stabilized vascular-related outcomes in patients with RA.
12	Pérez-Sánchez *et al.*, 2017 ^44^	Spain	Case–control study (n=146)	To evaluate the role of NETosis in the development of CVD in RA.	Patients with RA demonstrated enhanced (neutrophil extracellular trap) NET formation (p=0.02).	NETosis-derived products have a role in atherosclerosis and these products could be used to assess therapeutic response in RA patients.
13	Anghel *et al.*, 2021 ^45^	Romania	Retrospective observational study (n=115)	To assess the role of anti-TNFα drugs on cIMT.	After 1 year of anti-TNFα drugs, patients with RA showed a significant decrease in cIMT (p<0.001) and homocysteine levels.	This study concluded that biological treatments such as anti-TNF-α drugs are effective in reducing the cardiovascular risk in RA.
14	Majka *et al.*, 2017 ^46^	USA	Prospective cohort study (n=6532)	To assess whether RA-related antibodies are independent risk factors for atherosclerosis in RA.	Rheumatoid factor (RF) and anti-cyclic citrullinated peptide antibodies (anti-CCP) were increased in women with RA with coronary artery calcium (CAC) ≥300 (odds ratio 2.4 [95% CI 1.2–5.1] and odds ratio 4.1 [95% CI 1.3–12.7] respectively).	RA-autoantibodies are an independent risk factor for the development and propagation of subclinical and clinical atherosclerosis, indicating their role in atherogenesis in RA patients.
15	Rodríguez-Carrio *et al.*, 2014 ^47^	Spain	Prospective study (n=255)	To assess the role of EPC populations in RA.	Long-standing RA was correlated with a reduction in endothelial progenitor cell (EPC) population (p<0.001).	EPC imbalance is associated with an increased risk of CVD in RA and this association may be due to defective endothelial repair.
16	Nowak *et al.*, 2016 ^48^	Poland	Observational study (n=61)	To investigate the role of non-traditional risk factors in mediating atherosclerosis in RA.	RA patients had a higher level of oxidized low-density lipoprotein (oxLDL) compared to controls (p=0.04) and the presence of anti-CCP antibodies were associated with a greater cIMT (p=0.04).	The presence of anti-CCP antibodies and the oxLDL fraction can be determinants of CVD risk in RA.
17	Adawi *et al.*, 2018 ^49^	Israel	Prospective cohort study (n=57)	To assess endothelial function in patients with RA.	Out of 44 patients with RA, only 6 (13.6%) had normal endothelial function. RF was positive in 26 subjects (59.1%), whereas anti-CCP antibodies were found in 25 (56.8%) patients.	This study acknowledges the presence of accelerated atherosclerosis in RA and proposes the clinical use of FMD% as a measure of endothelial function to predict subsequent atherosclerosis.
18	Dimitroulas *et al.*, 2017 ^50^	UK	Prospective study (n=197)	To evaluate whether ADMA and ADMA levels are associated with atherosclerosis in RA.	There was a significant association between acetylcholine (Ach) and both SDMA (p=0.014) and ADMA:SDMA ratio (p=0.027), especially in the group with a higher inflammatory status.	Along with being novel biomarkers of endothelial dysfunction, SDMA and ADMA may also cause endothelial injury in patients with RA due to being mediators of inflammation.
19	Spinelli *et al.*, 2017 ^51^	Italy	Case–control study (n=80)	To assess whether anti-CarP antibodies are associated with atherosclerosis in RA.	There was a statistically significant association of anti-carbamylated protein antibodies (anti-CarP) with FMD in RA patients (p=0.04).	This study concluded that patients with RA experience endothelial dysfunction without the presence of traditional CVD risk factors.
20	González-Juanatey *et al.*, 2011 ^52^	Spain	Prospective study (n=118)	To assess endothelial function and cIMT in RA patients.	Patients with longer RA disease duration had higher cIMT values (p<0.001) and lower FMD (p<0.001), indicating increased cardiovascular risk.	Irrespective of CVD, endothelial dysfunction and cIMT were shown to increase as the time course of RA progressed.
21	Rodríguez-Carrio *et al.*, 2015 ^53^	Spain	Comparative study (n=194)	To assess RDW in relation to endothelial repair failure in RA patients.	An increase in red cell distribution width (RDW) caused a decrease in EPC counts in patients with established RA after adjusting for disease duration and traditional CVD risk factors (p<0.009).	RDW is associated with lower EPC populations and endothelial dysfunction and repair failure, thus contributing to the pathophysiology of CVD in RA.
22	Karpouzas *et al.*, 2020 ^54^	USA	Prospective cohort study (n=101)	To evaluate the role of inflammation in mediating atherosclerosis in RA.	Plaque burden was increased in 48% of patients and was associated with age, higher inflammation, and prednisone dose (p<0.05).	This study concluded that inflammation is an independent risk factor for subsequent premature atherosclerosis in RA.
23	Huaranga *et al.*, 2018 ^55^	Spain	Cross-sectional study (n=119)	To investigate the role of RA disease activity in the development of atherosclerosis.	High-risk patients, as defined by age, gender, blood pressure, presence of anti-cyclic citrullinated peptide antibodies and high disease activity, had a 3× increased risk of developing atherosclerotic plaques (p=0.037).	This study found that moderate or high disease activity is significantly associated with an increased risk of RA-related subclinical atherosclerosis.
24	Dehghan *et al.*, 2015 ^56^	Iran	Case–control study (n=70)	To investigate the prevalence of atherosclerosis in patients with inactive RA.	RA patients presented with more carotid plaques and a higher cIMT compared with the control group (48.6 *versus* 14.3% and 0.705±0.140 *versus* 0.580±0.125 respectively).	In patients with inactive RA, chronic inflammation is predictive of atherosclerosis.
25	Lo Gullo *et al.*, 2014 ^57^	Italy	Comparative study (n=66)	To assess the endothelial function and CPC populations in RA.	RA patients were found to have lower circulating progenitor cells (CPCs) compared to controls (p<0.001) and higher levels of ROS compared with controls (p<0.001).	Accelerated atherosclerosis and endothelial dysfunction in RA may be due to inflammation and oxidative stress caused by a reduction in CPCs.
26	Sahari *et al.*, 2014 ^58^	Malaysia	Case–control study (n=80)	To assess cIMT in RA patients without traditional CV risk factors.	RF antibodies and an active disease status were significantly correlated with higher cIMT values as compared with healthy controls (p=0.03 and p=0.02, respectively).	RA patients have a higher co-occurrence of premature atherosclerosis despite the absence of traditional CVD risk factors.
27	del Rincón *et al.*, 2015 ^59^	USA	Comparative study (n=487)	To investigate factors that mediate accelerated atherosclerosis in RA.	Increase in cIMT was significantly associated with ESR (Odds ratio 1.12 per 10 mm/h, [95% CI 1.02–1.23]) and the presence of CVD risk factors (Odds ratio 1.27 per risk factor, [95% CI 1.01–1.61]).	This study concluded that abnormal thickening of the cIMT in patients with RA was associated with chronic inflammation and the presence of traditional CVD risk factors.
28	Södergren *et al.*, 2019 ^60^	Sweden	Prospective cohort study (n=111)	To assess whether inflammatory biomarkers are associated with atherosclerosis in RA.	RA patients had significantly elevated levels of inflammatory biomarkers and pro-inflammatory cytokines such as MIC-1, TNF-R2, ICAM-1, VCAM-1 and endostatin compared with controls.	Inflammatory biomarkers may be involved in the development of premature subclinical atherosclerosis in patients with RA.
29	Södergren *et al.*, 2015 ^61^	Sweden	Prospective cohort study (n=111)	To assess the role of traditional CV risk factors and inflammation in mediating atherosclerosis in RA.	Patients had higher cIMT and poorer FMD values five years after being diagnosed with RA (p<0.01).	Both traditional CVD risk factors and inflammatory load are predictive of accelerated atherosclerosis in patients with RA.
30	Mohan *et al.*, 2014 ^62^	India	Case–control study (n=64)	To assess RA-related atherosclerosis in adult south Indian patients.	RA patients had a higher mean cIMT compared to healthy age and gender-matched controls (p=0.001).	cIMT is a useful marker to assess progression of atherosclerosis in patients with RA.
31	Agca *et al.*, 2021 ^63^	The Netherlands	Prospective study (n=89)	To assess the existence of arterial wall inflammation in RA patients.	A higher 18F-FDG uptake was detected in the wall of the carotid arteries (p<0.01) and the aorta (p<0.01) in patients with RA, even after adjusting for CVD risk factors.	This study concluded that arterial wall inflammation is implicated in the pathophysiology of RA-related vascular complications.
32	Yamamoto *et al.*, 2019 ^64^	Japan	Prospective study (n=454)	To determine risk factors for the occurrence of atherosclerosis in RA patients.	238 (52%) RA-patients had atherosclerosis. Traditional CVD risk factors including age, hypertension, and total/HDL cholesterol ratio were significantly associated with cIMT (p<0.01).	Inflammatory mediators involved in the RA disease process influence the development of atherosclerotic plaques so the management of RA should involve controlling CVD risk factors and disease activity.
33	Guin *et al.*, 2013 ^65^	India	Prospective study (n=35)	To assess whether DMARDs improve vascular function in RA patients.	cIMT value decreased after 1 year of DMARD treatment (p=0.002).	DMARDs are useful in the management of RA and halting the progression of RA-related atherosclerosis.
34	Dalbeni *et al.*, 2017 ^66^	Italy	Prospective study (n=256)	To assess risk-factors that accelerate atherosclerosis in RA.	Among non-modifiable CVD risk-factors, age was significantly associated with carotid segmental distensibility, cIMT, and atherosclerotic plaques in RA patients (p<0.001).	Age is implicated as the major determinant of premature atherosclerosis in arthritis.
35	Ajeganova *et al.*, 2012 ^67^	Sweden	Prospective study (n=105)	To assess the influence of atherosclerosis and disease activity in development of CVD.	Improvement in DAS28 and usage of methotrexate (MTX) reduced the risk of a CVD event [hazard ratio 0.68 (95% CI 0.5–0.97) and 0.34 (95% CI 0.12–0.91), respectively].	Elevated levels of oxLDL may contribute to the pathogenesis of chronic inflammation. Controlling disease activity and early treatment of RA is pivotal in improving vascular-related outcomes in RA patients.
36	Liu *et al.*, 2017 ^68^	China	Prospective study (n=85)	To investigate the progression of vascular calcification in RA patients.	Coronary calcium score (CS) was found to be significantly increased in the coronary artery (p<0.01), carotid artery (p<0.01), and aorta (p<0.01).	This study concluded that there is a significant increase in vascular calcification over 10 years following RA diagnosis.
37	Udachkina *et al.*, 2018 ^69^	Russia	Prospective study (n=74)	To assess CAC in patients with RA.	Coronary artery calcification (CAC) was detected in 34 (46%) RA patients. In RA patients who had ischemic heart disease, occurrence of CAC was 100%.	This study found that CAC was highly prevalent in patients with early RA and this association was positively correlated with age.
38	Tuzcu *et al.*, 2019 ^70^	Turkey	Case–control study (n=69)	To evaluate whether endocan can be used as a marker for endothelial dysfunction in RA patients.	RA patients had significantly higher values of human endothelial cell-specific molecule-1 (endocan) compared to age and sex-matched controls (p=0.009).	Endocan can be used as a biomarker to assess endothelial dysfunction and atherosclerotic plaques in patients with RA.
39	Fan *et al.*, 2012 ^71^	China	Case–control study (n=148)	To assess the presence of atherosclerosis in RA patients using cIMT and FMD%.	Patients with RA had a significantly higher cIMT compared to the control group (p<0.001) and significantly lower brachial artery FMD% than controls (p<0.001).	This study concluded that RA patients had subclinical atherosclerosis as assessed by cIMT and FMD%.
40	Yiu *et al.*, 2011 ^72^	Hong Kong	Comparative study (n=170)	To assess the prevalence of vascular calcification in patients with RA.	Aortic valve calcification (AVC) and mitral valve calcification (MVC) was highly prevalent in patients with RA or systemic lupus erythematosus (SLE) (p<0.01).	MVC was an independent risk factor for premature atherosclerosis in patients with RA or SLE after adjusting for clinical parameters.
41	Lo Gullo *et al.*, 2014 ^73^	Italy	Comparative study (n=50)	To assess the impact of inflammation in mediating atherosclerosis in RA.	RA patients had a lower count of CD34+ cells, but higher expressions of ROS, TLR3 and IL-1β compared to the control group. RA patients also had elevated levels of CRP, suggesting a proinflammatory milieu.	Accelerated atherosclerosis in RA is mediated by RA-related inflammatory changes in the vasculature.
42	Chung *et al.*, 2013 ^74^	USA	Prospective study (n=990)	To assess risk-factors associated with CAC in RA.	There was no statistical significance in the incidence of coronary artery calcium (CAC) in RA patients compared to controls (p=0.68).	Inflammatory markers were not associated with development of CAC in RA patients. Traditional CVD risk factors, rather than inflammation, are associated with CAC in RA.
43	Vázquez-Del Mercado *et al.*, 2015 ^75^	Mexico	Cross-sectional study (n=82)	To assess the impact of anti-CCP antibodies on cIMT in RA patients.	RA patients with anti-CCP positive antibodies had a significantly increased cIMT compared to the control group and anti-CCP negative group (p<0.001).	Increased incidence of atherosclerosis and CVD events in RA patients are associated with anti-CCP and CRP levels.
44	Di Minno *et al.*, 2015 ^76^	Italy	Meta-analysis (n=1688)	To assess endothelial function in RA patients using FMD.	Patients with RA had a lower FMD compared to controls (p=0.0003), but no significant difference was found in nitrate-mediated dilation (NMD) (p=0.49).	This meta-analysis concluded that patients with RA should be monitored for endothelial dysfunction and therapeutic strategies must be planned to prevent vascular damage in these individuals.
45	Di Franco *et al.*, 2012 ^77^	Italy	Prospective study (n=40)	To assess the role of SDMA and ADMA in mediating endothelial dysfunction in RA patients.	In patients with early RA, ADMA levels were higher than in the control group (p=0.007). After treatment with DMARDs, ADMA levels were significantly decreased in this group (p=0.012).	This study concluded that asymmetric-dimethylarginine and apelin can be used as markers of endothelial impairment in RA patients.
46	Kerekes *et al.*, 2011 ^78^	Hungary	Prospective study (n=8)	To assess the effect of TNF-α inhibitors on vascular function in RA patients.	In patients treated with adalimumab, there was a significant decrease in CRP levels (p=0.04) and DAS28 (p<0.0001). Endothelial function, as measured by FMD also improved (p<0.05).	Treatment with TNF-α inhibitors improved endothelial function and ameliorated atherosclerosis in patients with early RA, suggesting that TNFα is a key mediator of premature atherosclerosis in RA.
47	Wahlin *et al.*, 2016 ^79^	Sweden	Prospective study (n=22)	To evaluate factors that are associated with CAC in RA patients.	Patients with RA who had coronary artery calcification (CAC) were found to have increased values of DAS28 and ESR (p<0.01 and p<0.01, respectively).	In patients with long-standing RA, inflammation is a key mediator of CAC.
48	Ahmed *et al.*, 2016 ^80^	Norway	Prospective study (n=39)	To assess the role of inflammatory molecules in mediating atherosclerosis in RA.	In patients with RA, TNF was found in 63% of biopsy specimens whereas only 30% of specimens from the control group expressed TNF (p=0.04).	Proinflammatory cytokines are involved in the pathophysiology of mediating accelerated atherosclerosis in RA.
49	Im *et al.*, 2015 ^81^	Korea	Prospective study (n=615)	To assess inflammatory burden in RA patients.	RA patients had a higher mean cIMT compared to controls (p<0.001). Plaques were more common RA patients (74%) than in controls (26%) (p=0.004).	Inflammatory mediators and traditional CVD risk factors act synergistically to contribute to the development of premature atherosclerotic disease in RA.
50	Profumo *et al.*, 2012 ^82^	Italy	Prospective study (n=62)	To assess biomarkers that can be used to predict atherosclerosis in RA.	RA patients had higher levels of ox-LDL compared to controls. RA patients also had lower levels of NO compared to healthy subjects (p<0.0001).	This study concluded that ox-LDL and NO are biomarkers of premature atherosclerotic disease in RA.
51	Chandrasekharan *et al.*, 2018 ^83^	USA	Cross-sectional study (n=357)	To assess the role of SDMA and ADMA in mediating CVD in RA patients.	Patients with RA had elevated levels of ADMA (p<0.001) and SMDA (p<0.001) and lower levels of nitric oxide synthase (NOS) (p<0.001) compared with healthy age- and sex-matched controls.	This study concluded that dysregulated L-arginine metabolism may contribute to the pathophysiology of developing CVD in RA.
52	Okano *et al.*, 2017 ^84^	Japan	Prospective cohort study (n=413)	To assess whether anti-CCP antibodies contribute to atherosclerosis in RA.	Carotid plaque was found more commonly in RA patients than control subjects (p=0.027). RA patients with plaques had higher levels of anti-CCP antibodies compared to RA patients without plaques (p=0.005).	High serum levels of anti-CCP antibodies are associated with causing severe atherosclerotic plaque in RA.
53	Geraldino-Pardilla *et al.*, 2017 ^85^	USA	Prospective study (n=195)	To assess the role of ACPAs in mediating CAC in RA.	In patients with RA, high levels of ACPAs were positively correlated with higher CAC scores (p=0.001) and this association was observed even after adjusting for traditional CVD and RA risk factors (p=0.03).	ACPAs play a significant role in the pathophysiology of atherosclerosis in RA.
54	Marder *et al.*, 2011 ^86^	USA	Prospective study (n=51)	To evaluate whether IL-17 is involved in mediating endothelial dysfunction in RA patients.	Patients with RA who had anti-CCP antibodies in the serum had higher levels of IL-17 compared to patients who were anti-CCP negative (p=0.01).	IL-17 participates in the development and propagating of endothelial impairment and CVD in patients with RA.
55	Barbarroja *et al.*, 2014 ^87^	Italy	Prospective study (n=106)	To assess the role of RA-derived antibodies in mediating atherosclerosis in RA.	In RA patients, anti-CCPs antibody levels were significantly associated with age and DAS28 (p=0.024 and p=0.001, respectively). RA patients who were anti-CCP positive were found to have a strong association with cIMT (p<0.01).	This study concluded that autoantibodies such as anti-CCPs are significantly associated with the proatherogenic milieu seen in RA patients.
56	Davies *et al.*, 2021 ^88^	UK	Cross-sectional study (n=182)	To assess the role of IL-6 in mediating atherosclerosis in RA.	In patients with established RA, a positive correlation was found between the DAS28 index and sVCAM-1 (p = 0.017).	This study concluded that sVCAM-1 is a risk-factor for the progression of atherosclerosis in patients with RA and that IL-6 plays a role in mediating this.
57	van Breukelen-van der *et al.*, 2015 ^89^	The Netherlands	Cross-sectional study (n=360)	To assess cIMT values in RA patients and determine the clinical implications of this.	There was no association between RA and cIMT. In RA patients, cIMT was associated with age and systolic blood pressure (p<0.001) and (p=0.003).	This study concluded that in RA patients with low disease activity, there is no significant association with cIMT, suggesting that therapeutic strategies that target CVD risk factors seem to improve cardiovascular risk in RA.
58	Karpouzas *et al.*, 2014 ^90^	USA	Prospective study (n=300)	To assess the prevalence of CAC in RA patients.	RA patients had higher CAC compared with controls (p < 0.0001). Atheroscletic plaques were more frequently found in patients with RA compared to the control cohort (71% *versus* 45%, p<0.0001).	This study concluded that RA patients without any known CVD had a higher occurrence and severity of coronary atherosclerotic plaque.
59	Kassem *et al.*, 2011 ^91^	Egypt	Prospective study (n=30)	To assess the importance of non-traditional risk factors in mediating atherosclerosis in RA.	There was an association between RA patients who harbored carotid atherosclerosis and inflammatory markers such as CRP*, ESR* and IL-6** and VCAM-1*, a marker of endothelial impairment (*p<0.001 and **p<0.05).	This study found a significant correlation between atherosclerosis and inflammatory markers, endothelial dysfunction, and rheumatoid arthritis related antibodies.
60	Wahab *et al.*, 2015 ^92^	Egypt	Prospective study (n=75)	To assess whether anti-CCP antibodies are involved in mediating atherosclerosis in RA.	RA patients also had higher levels of anti-CCP antibodies compared to controls (p=0.001).	Anti-CCP antibodies are a useful indicator of subclinical atherosclerosis in patients with RA.
61	Mondal *et al.*, 2011 ^93^	India	Prospective study (n=146)	To assess endothelial function in RA patients.	RA patients had a lower FMD value than controls (p<0.001). There was a negative association between FMD and CRP levels (p<0.01).	RA patients without any known CVD risk factors have altered endothelial function and an increased susceptibility to premature atherosclerosis.
62	Arida *et al.*, 2021 ^94^	Greece	Prospective study (n=85)	To investigate whether PCSK9 or LDLR levels are associated with subclinical CVD in RA patients.	PCSK9 was significantly associated with atheromatous plaques (p=0.033) and LDLR concentration was also correlated with plaque presence (p=0.005) in RA patients.	The PCSK9/LDLR system was found to be associated with subclinical atherosclerosis in RA.
63	Mena-Vázquez *et al.*, 2022 ^95^	Spain	Observational study (n=160)	To analyze association of postprandial lipidemia with subclinical atherosclerosis in RA.	Postprandial hyperlipidemia (PPHL) in RA patients was associated with subclinical atherosclerosis (p=0.037), TNF-α (p=0.048), and high-sensitivity C-reactive protein (p=0.027).	PPHL in RA is associated with inflammation and subclinical atherosclerosis.
64	Wahlin *et al.*, 2021 ^96^	Sweden	Prospective study (n=79)	To examine whether regulators of bone formation or turnover are associated with RA-related atherosclerosis.	Osteocalcin (OCN) and osteoprotegerin (OPG) were significantly associated with IMT after 11 years (p=0.03).	Markers of bone turnover were associated with IMT in RA patients.
65	Beyazal *et al.*, 2016 ^97^	Turkey	Retrospective cohort study (n=116)	To assess whether serum OPG levels are associated with arterial stiffness and cIMT in RA patients.	OPG levels were higher in RA patients than controls (p < 0.001) and were significantly associated with cIMT.	OPG may be a useful marker to assess CV risk in RA patients.
66	Esaily *et al.*, 2021 ^98^	Egypt	Case–control study (n=200)	To examine the relationship between the cellular communication network factor 1 (CCN1) and RA-induced atherosclerosis.	CCN1 was positively correlated with cIMT in RA patients (p<0.001).	Disruptions in serum CCN1 levels are associated with subclinical atherosclerosis in RA patients.
67	Mulumba *et al.*, 2019 ^99^	Democratic Republic of Congo	Cross-sectional study (n=75)	To describe the prevalence of subclinical atherosclerosis in RA patients.	Risk factors associated with subclinical atherosclerosis in RA patients included being a woman aged >55 years (p=0.028), DAS28-ESR >2.6 (p=0.044), severe RA (p=0.035) and obesity (p=0.026).	Subclinical atherosclerosis is highly prevalent in RA patients.
68	Elshereef *et al.*, 2015 ^100^	Egypt	Retrospective study (n=152)	To evaluate prevalence of atherosclerosis in RA patients and correlate it with disease activity.	31.3% of asymptomatic RA patients had atherosclerosis compared with 5% control subjects (p=0.003).	Long-term RA patients have a high frequency of subclinical atherosclerosis.
69	Ristić *et al.*, 2015 ^101^	Serbia	Cross-sectional study (n=74)	To determine whether von Willebrand factor (vWF) activity is associated with subclinical atherosclerosis in RA patients.	vWF activity was higher in RA patients with subclinical atherosclerosis (p<0.05) or atherosclerotic plaques (p<0.05).	vWF is a valuable biomarker to determine premature atherosclerosis in RA patients.
70	Södergren *et al.*, 2015 ^102^	Sweden	Prospective study (n=71)	To assess whether the level of Lp-PLA2 is associated with subclinical atherosclerosis.	Lp-PLA2 was significantly associated with IMT at T0 and T5 and flow mediated dilation (FMD) (p<0.05).	Lp-PLA2 is associated with both subclinical atherosclerosis and disease severity in RA patients.
71	Mena-Vázquez *et al.*, 2020 ^103^	Spain	Observational study (n=80)	To analyze association of postprandial lipidemia with subclinical atherosclerosis in RA.	In RA patients, cIMT was associated with postprandial ApoB48 (OR (95% CI), 1.15 (1.0-1.3)) and total ApoB (OR [95% CI], 1.12 [1.1–1.2]).	Atherogenic particles such as ApoB48 are associated with endothelial dysfunction in RA patients.
72	Södergren *et al.*, 2010 ^104^	Sweden	Prospective study (n=123)	To assess the presence of premature atherosclerosis in patients with very early RA.	RA patients had a significantly increased IMT and higher levels of VWF, sICAM-1 (p<0.05), and MCP-1 (p=0.001) compared with controls.	IMT and FMD are related to biomarkers associated with endothelial dysfunction and atherosclerosis in RA patients.
73	Barbara *et al.*, 2018 ^105^	Brazil	Prospective study (n=180)	To investigate the association of serum MBL levels and its association with IMT in RA patients.	RA patients had a significantly lower MBL serum concentration in relation to controls (528 ng/mL *versus* 937.5 ng/mL, p= 0.05, respectively).	RA patients had lower MBL serum levels than controls. MBL was not associated with disease activity, ESR, autoantibodies, or IMT.

## Discussion

### The role of inflammatory cytokines

The pathophysiology of accelerated atherosclerosis in RA is due to a complex interplay between various proinflammatory mediators. TNF-α is an inflammatory cytokine that is highly elevated in the synovial fluid of individuals with RA and is involved in mediating premature atherogenesis by complex signaling pathways involving the p38 mitogen-activated protein kinase (MAPK) and the transcription factor nuclear factor-κB (NF-κB). ^11^^,^^106^ As described earlier, TNF-α acts synergistically with IL-6 to form lipid-laden macrophages (foam cells), which are prototypic in the development of atherosclerotic plaques. ^13^^–^^16^ The mechanistic pathway that contributes to this process involves the upregulation of the human scavenger receptor-A (SR-A) and lectin-like oxidized LDL receptor-1 (LOX1) on macrophages. ^15^ Anti-TNF-α therapy has become a mainstay for treatment of RA. ^106^^,^^107^ In a prospective study by Végh *et al.*, anti-TNF-α therapy was shown to stabilize vascular-related outcomes in RA patients by ameliorating endothelial function. ^43^ Similar findings were also reported by other studies included in this review, where TNF-α inhibition was found to significantly improve vascular function in RA patients. ^42^^,^^45^^,^^78^

Davies *et al.* found a positive correlation between RA disease activity and sVCAM-1, and associated it with the role of IL-6 in mediating atherosclerosis in RA. ^88^ They went on to conclude that IL-6 trans-signaling plays an important role in vascular dysfunction in RA and blocking this pathway may be useful for RA patients. IL-1 is considered to be a proatherogenic cytokine as it stimulates SMC proliferation by autocrine induction of the platelet-derived growth factor (PDGF). ^20^^,^^108^ SMCs contribute to the initiation of atherosclerosis by producing an extracellular matrix (ECM), precipitating lipid uptake, and inducing foam cell formation to ultimately form a fibrous cap within the vessel wall. ^109^ IL-1 also induces its own gene expression, thus creating a powerful positive feedback loop to maintain a proatherogenic milieu. ^20^ Lo Gullo *et al.* reported that RA patients had a higher expression of IL-1β and elevated levels of C-reactive protein (CRP) compared with the control group, suggesting a proinflammatory milieu mediated by this cytokine ^73^ Kassem *et al.* assessed the role of non-traditional risk factors and found that RA patients with carotid atherosclerosis harbored inflammatory markers such as CRP, ESR, IL-6, and VCAM-1. ^91^ Similar findings were reported by Karpouzas *et al.* and Agca *et al.* who concluded that inflammation is implicated in the pathophysiology of RA-related vascular complications. ^54^^,^^63^ Other studies also found significantly elevated levels of inflammatory biomarkers and pro-inflammatory cytokines in RA patients, further supporting the role of cytokines in premature atherosclerosis in RA. ^60^^,^^80^ Individuals with RA have elevated circulating levels of IL-17 as a result of T-helper 17 (Th17) cell activation and differentiation. ^10^^,^^11^^,^^110^ Although the pathomechanisms that implicate IL-17 as a mediator of atherogenesis in the setting of RA remain unclear, Marder *et al.* proposed that IL-17 may disrupt normal endothelial function, stimulate myocardial fibrosis, and lower arterial compliance, thus promoting atherosclerosis. ^86^ Yamamoto *et al.* reported that both traditional CVD risk-factors and inflammatory mediators influence the development of atherosclerosis in RA patients and concluded that the management of RA should involve controlling CVD risk factors and RA disease activity. ^64^ A wealth of data shows that the role of inflammatory cytokines is pivotal when considering the pathomechanisms that explain the increased incidence of premature atherosclerosis in RA.

### The role of neutrophil extracellular traps

The role of NETs in autoimmune disorders is explained by the process of NETosis, which is described as an imbalance between NET formation and NET breakdown. ^10^ In patients with RA, NET formation is thought to be triggered by autoantibodies and immunostimulatory molecules. ^21^ NETs contain citrullinated vimentin (cVim), which is involved in secreting proinflammatory cytokines such as TNF-α and IL-1, thus continually maintaining a state of inflammation in RA. ^21^^,^^111^ Even though the role of neutrophils in mediating atherosclerosis has been historically denied, recent evidence shows that neutrophils are involved in progressive endothelial damage, recruitment of proinflammatory monocytes, and foam cell formation. ^24^^–^^26^ Researchers have detected the presence of NETs in atherosclerotic plaques of both humans and mice, which may contribute to the pathophysiology of RA-related atherosclerosis. ^23^^,^^112^^,^^113^ Pérez-Sánchez *et al.* found that RA patients had enhanced NETosis, which correlated with disease activity and inflammatory and oxidative profiles in these patients. They concluded that NETosis-derived products play a role in mediating atherosclerosis in RA and may be used as a diagnostic tool. ^44^ The lack of primary research studies on this topic area make it difficult to draw definitive conclusions about the role NETosis in RA and atherosclerosis. More research in this particular field would be beneficial in evaluating the diagnostic potential of NETosis-derived products and assessing whether the inhibition of NETs would hold therapeutic value in RA.

### The role of RA-related autoantibodies

Citrullinated proteins within the synovia of patients with RA are a target for anti-citrullinated peptide antibodies (ACPAs). ^27^^,^^28^ Sokolove *et al.* found that citrullinated fibrinogen within atherosclerotic plaques are also targeted by RA-derived ACPAs. ^28^ Clavel *et al.* found that RA-specific ACPA immune complexes have the potential of inducing macrophage-driven TNF-α secretion via the Fc-gamma receptor IIa (FcγRIIa). ^114^ As detailed earlier, the contribution of TNF-α as an inflammatory mediator of atherosclerosis is remarkable, which provides evidence to support the pathophysiology of RA autoantibody-derived atherosclerosis. RA-derived autoantibodies like ACPAs were found in the serum of patients with RA and were considered an independent risk factor for the development of subclinical and clinical atherosclerosis. ^51^ Nowak *et al.* found that the presence of anti-CCP antibodies were associated with greater cIMT values, as also confirmed by Vázquez-Del Mercado *et al.* ^48^^,^^75^ Wahab *et al.* also found higher levels of anti-CCP antibodies in RA patients compared to controls and suggested its use as a useful indicator of subclinical atherosclerosis. ^92^ A number of studies included in this review found similar results concerning RA-derived autoantibodies and atherosclerosis. ^51^^,^^55^^,^^84^^,^^85^^,^^87^ An interesting phenomenon was reported by Jacobsen *et al.*, who found polymorphic variations in the mannose-binding lectin gene (MBL) were associated with high scores of RA disease activity, C-reactive protein-based DAS28, and physical disability in anti-CCP-positive RA patients. ^115^ MBL, a serum protein, plays an instrumental role in regulating innate immunity by binding to repeating sugar motifs to activate the complement system *via* MBL-associated serum proteases. ^116^ Barbara *et al.* found that RA patients had a significantly lower MBL serum concentration in relation to controls and found no statistically significant association between MBL and disease activity, ESR, autoantibodies, or IMT. ^105^ A cross-sectional study found that both high and low levels of MBL in RA patients were associated with an increased common carotid artery intima-media thickness (cc-IMT), indicating a quadratic U-shaped relation between serum MBL and ccIMT. ^117^ More studies would be valuable to assess whether MBL truly plays a role in mediating atherosclerosis in RA patients.

### The role of endothelial dysfunction

Atherosclerosis is a consequence of progressive endothelial damage and dysfunction. ^30^ The endothelium, as an organ, functions as an intimate vascular barrier that is involved with maintaining vascular tone, blood hemostasis, leukocyte migration, and antigen presentation, among other physiological processes. ^118^^,^^119^ Endothelial dysfunction is heavily involved in the pathogenesis of atherosclerosis by inducing cell adhesion molecules, facilitating leukocyte emigration, promoting cytokine production, platelet activation, and SMC proliferation ^120^ A number of studies have found elevated serum levels of asymmetric dimethylarginine (ADMA) in RA patients. ^121^^–^^124^ ADMA is a potent inhibitor of endothelial nitric oxide synthase (eNOS), an enzyme responsible for the synthesis of vasoprotective NO ^125^ NO serves as a cardioprotective molecule due to its vasodilatory properties and its ability to inhibit platelet aggregation, suppress adhesion molecules, maintain endothelial barrier integrity, and regulate SMC proliferation. ^126^

Di Franco *et al.* found that RA patients had elevated levels of ADMA and proposed its use as a biomarker of vascular endothelial dysfunction, as also concluded by Dimitroulas *et al.* and Chandrasekharan *et al.* ^50^^,^^77^^,^^83^ Endothelial progenitor cells (EPCs) have also been implicated in RA-related atherogenesis. ^10^ An inverse relationship between circulating EPCs and endothelial function has been established by numerous studies. ^127^^–^^129^ When considering RA, a reduction in EPCs has been reported, possibly due to C-reactive protein-mediated apoptosis of EPCs ^130^ Rodríguez-Carrio *et al.* reported long-standing RA was correlated with a reduction in EPCs and concluded that EPC imbalance is associated with endothelial dysfunction and subsequent CVD in RA. ^47^ Similar findings were also reported by other studies included in this review. ^53^^,^^57^ Progressive endothelial dysfunction along with a reduction in EPCs in RA results in the creation of a proatherogenic environment, thus facilitating atherosclerosis and CVD in affected individuals.

### Prevalence of accelerated atherosclerosis in RA

Carotid intima media thickness (cIMT) has popularly been used as a marker for subclinical atherosclerosis. In our review, a number of studies utilized cIMT to assess the prevalence of subclinical atherosclerosis in RA. In a cross-sectional study by Hannawi *et al.*, patients with RA had significantly higher cIMT values and a higher carotid plaque burden compared to healthy controls. ^38^ Krajnc *et al.* also found that patients with RA had a higher cIMT compared with controls, suggesting atherosclerotic plaque build-up and increased risk for subsequent CVD. ^39^ A plethora of other studies included in this review reported similar findings and concluded that there is a high prevalence of premature atherosclerosis in patients with RA. ^33^^,^^34^^,^^36^^,^^40^^,^^41^^,^^52^^,^^56^^,^^58^^–^^62^^,^^65^^,^^66^^,^^71^^,^^81^^,^^99^^,^^100^^,^^104^ However, in a cross-sectional study by van Breukelen-van der *et al.*, no significant association was found between RA and cIMT. These conflicting findings were attributed to the patients having low disease activity and well-controlled RA, suggesting therapeutic strategies that target CVD risk factors seem to improve overall cardiovascular risk in RA. ^89^

Flow-mediated dilation (FMD) is a non-invasive method to assess endothelial dysfunction and has been used as a predictor of early atherosclerosis. Verma *et al.* found that RA patients had lower FMD and higher cIMT compared to controls, consistent with endothelial dysfunction and accelerated atherosclerosis in patients with RA. ^37^ Adawi *et al.* also acknowledged the high prevalence of accelerated atherosclerosis in RA patients and proposed the clinical use of FMD as a measure of endothelial function to predict subsequent atherosclerosis. ^49^ Two other studies also reported similar findings regarding endothelial dysfunction in RA patients and the usefulness of FMD as an early marker of atherosclerosis. ^76^^,^^93^

Coronary calcium score (CS) utilizes computed tomography to detect the presence of vascular calcification. In a prospective study by Liu *et al.*, the CS was found to be significantly increased in the coronary artery, carotid artery, and aorta of RA patients. ^68^ In another prospective study, coronary artery calcification (CAC) was detected in 46% of RA patients. ^69^ Other studies also reported similar findings. ^72^^,^^90^ In a prospective study by Wahlin *et al.*, patients with RA who had CAC were found to have increased values of DAS28 and ESR, implying that inflammation plays a role in mediating RA-induced CAC. ^79^ Contrastingly, Chung *et al.* found no statistical significance in the incidence of CAC in RA patients compared to controls and concluded that traditional risk-factors for CVD, rather than inflammatory markers are responsible for CAC and atherosclerosis in RA. ^74^ This study also reported that once RA patients developed CAC, the rate of progression was found to be similar to the progression seen in control participants. This study acknowledges that their results were not concordant with their primary hypothesis, and one explanation offered was that the study focused on subclinical atherosclerosis, and RA patients with prior events, who may have contributed to a greater progression of CAC had been excluded, resulting in differential bias. Overall, there is abundant evidence to conclude that premature atherosclerosis in RA is highly prevalent, and that both traditional CVD risk-factors and inflammatory mediators play a role in mediating this process.

### Novel markers associated with accelerated atherosclerosis in RA

Postprandial hyperlipidemia (PPHL) has been shown to be an independent predictor of CVD. ^131^ Mena-Vázquez *et al.* found that PPHL in RA patients was significantly associated with subclinical atherosclerosis, TNF-α, and high-sensitivity C-reactive protein, suggesting that PPHL in RA is associated with inflammation and subclinical atherosclerosis. ^95^ Other studies have also found that cIMT is associated with postprandial ApoB48 and total ApoB, providing evidence that atherogenic chylomicron remnants contribute to atherosclerosis in RA. ^103^^,^^132^

Biomarkers associated with bone turnover such as osteoprotegerin (OPG) have been associated with CAC, carotid plaque, and IMT. ^133^^–^^135^ Wahlin *et al.* examined the role of bone turnover markers in mediating atherosclerosis in RA and found that Osteocalcin (OCN) and OPG were significantly associated with IMT after 11 years, especially in patients with joint erosions. However, there was no significant association between collagen markers of *ongoing* bone turnover and IMT, suggesting that bone turnover and atherosclerosis may have different pathogenic mechanisms in the setting of RA. ^96^ Beyazal *et al.* also found higher OPG levels in RA patients than controls and concluded that OPG may be a useful marker to assess CV risk in RA patients. ^97^ More studies investigating this topic area may be beneficial to clarify whether bone turnover truly plays a role in premature atherosclerosis in RA.

Other novel markers found to be associated with accelerated atherosclerosis in RA patients were von Willebrand factor (vWF), ^101^ cellular communication network factor 1 (CCN1), ^98^ human endothelial cell-specific molecule-1 (endocan), ^70^ PCSK9/LDLR system, ^94^ and oxLDL. ^67^^,^^82^ Lipoprotein-associated phospholipase A _2_ (Lp-PLA _2_) is a biomarker used to assess vascular inflammation. ^136^ Södergren *et al.* found that Lp-PLA2 is associated with both subclinical atherosclerosis and disease severity in RA patients, ^102^ however, Bes *et al.* found no significant difference between Lp-PLA2 enzyme in RA patients and healthy controls, possibly because RA patients were undergoing treatment and had low disease activity scores. ^35^ Similarly, there are other novel biomarkers being investigated in their role in developing accelerated atherosclerosis in the setting of RA. More studies in this topic area may be beneficial to predict premature atherosclerosis in RA and identify new therapeutic targets.

### Limitations

Due to the rigorous inclusion and exclusion criteria, studies that may contain supportive evidence according to their results were not included because their aim was not in line with our search criteria. Moreover, some studies did not explicitly state information regarding the study setting and type of study. Based on the current literature and evidence, there are various pathophysiological processes involved in accelerating atherosclerosis in RA which made it difficult to draw definitive conclusions on whether inflammation or traditional cardiovascular risk-factors work together or independently to contribute to these findings. 

## Conclusion

In conclusion, abundant evidence exists to support a high prevalence of accelerated atherosclerosis among RA patients. Since cardiovascular morbidity and mortality in RA is strikingly high, it is important to understand the mechanisms that initiate and govern atherosclerosis in RA so tailored therapeutic regimens can be developed to reduce the CV burden that RA patients carry. We found evidence to support the role of proinflammatory cytokines, neutrophil extracellular traps, RA-related autoantibodies, and endothelial dysfunction in the pathophysiology of RA-mediated atherosclerosis. Proinflammatory cytokines such as IL-6 and TNF-α are involved in the formation of atherogenic foam cells. RA-derived autoantibodies are involved in exacerbating the inflammatory potential of macrophages and endothelial dysfunction is involved in disrupting the integrity of the vascular barrier which create a proatherogenic milieu and favour subsequent atherosclerotic disease. The question of whether inflammation and traditional CV risk factors work synergistically to produce atherosclerosis in RA or if one is more significant than the other remains. Nevertheless, therapies targeting both the inflammatory load or traditional CV risk-factors seem to improve vascular outcomes in RA patients. Lastly, novel markers of atherosclerosis in RA may be useful in predicting early atherosclerosis and serve as new targets for pharmacological intervention.

## Data availability

No data are associated with this article.

## Reporting guidelines

Raj, Rhea; Gorantla, Vasavi; Thomas, Sneha (2022): Accelerated atherosclerosis in rheumatoid arthritis: a systematic review. figshare. Dataset. https://doi.org/10.6084/m9.figshare.19618947.v1

## Ethical statement

Not applicable. No patient data or animal studies were used in this review.

## Consent from patients

In this review, no patient information was used. Consent is not applicable.
