# Supplementary material for: Accelerated atherosclerosis in rheumatoid arthritis: a systematic review
Source: F1000Res. 2023 Jan 16;11:466. Originally published 2022 Apr 27. [Version 2] doi: 10.12688/f1000research.112921.2 (PMC9551388; doi:10.12688/f1000research.112921.2)
Supplement: Peer review history

# Peer review: Reviewer response for version 2

**DOI:** 10.5256/f1000research.143076.r160321

**Published:** 2023-01-17

**Authors:** Syed-Rehan Hussain

**Affiliations:** 1 Division of Allergy & Immunology, Center for Clinical and Translational, Abigail Wexner Research Institute, Nationwide Children's Hospital, Columbus, OH, USA

I have looked at the revised manuscript and the authors have addressed all my concerns therefore for I approve the revised version the article.

Are the rationale for, and objectives of, the Systematic Review clearly stated?

Yes

Is the statistical analysis and its interpretation appropriate?

Not applicable

Are sufficient details of the methods and analysis provided to allow replication by others?

Partly

Are the conclusions drawn adequately supported by the results presented in the review?

Partly

Reviewer Expertise:

NA

I confirm that I have read this submission and believe that I have an appropriate level of expertise to confirm that it is of an acceptable scientific standard.

# Peer review: Reviewer response for version 2

**DOI:** 10.5256/f1000research.143076.r160322

**Published:** 2023-01-17

**Authors:** Praveen Kumar Kodumuri

**Affiliations:** 1 Department of Physiology, TomoRiba Institute of Health & Medical Sciences, Naharlagun, Arunachal Pradesh, India

Dear Authors,

 I'm satisfied with the amendments, and I recommend the manuscript for publication.

Are the rationale for, and objectives of, the Systematic Review clearly stated?

Yes

Is the statistical analysis and its interpretation appropriate?

Not applicable

Are sufficient details of the methods and analysis provided to allow replication by others?

Yes

Are the conclusions drawn adequately supported by the results presented in the review?

Yes

Reviewer Expertise:

Neuroscience, Cardiology, Electrophysiology and Pulmonology

I confirm that I have read this submission and believe that I have an appropriate level of expertise to confirm that it is of an acceptable scientific standard.

# Peer review: Reviewer response for version 1

**DOI:** 10.5256/f1000research.124651.r150874

**Published:** 2022-10-10

**Authors:** Praveen Kumar Kodumuri

**Affiliations:** 1 Department of Physiology, TomoRiba Institute of Health & Medical Sciences, Naharlagun, Arunachal Pradesh, India

Authors have done exhaustive review of various research articles in order bring the possible link/s between augmented atherosclerosis in rheumatoid arthritis. This review article provides the information about mechanisms, risk factors that initiate and hastens the atherosclerosis in rheumatoid arthritis.

 I have following comments/suggestions for the author’s consideration:

1. Authors may provide precise details of “how 73 research articles were considered among 21,235”. This may clear the ambiguity of selection.
2. In exclusion criteria, authors may be asked to provide clarity of information. Overall authors may be asked to provide clear information on inclusion and exclusion criteria. Selection criterion lacks clarity.
3. Some of the abbreviations may be provided with expanded form like TNF, IL, LDL, sVCAM, SMC, ESR and CVD etc.
4. In the “Discussion” part, authors have mentioned that “Anti-TNF-α therapy and vascular related outcomes” and this may be avoided as it is out of review article purview (or) authors may provide rationale for mentioning the specific details.
5. “Conclusion” of the review article is more elaborate, and authors may be asked to provide in concise form.

Authors are to be commended for their extensive research and careful analysis of various research articles.

Are the rationale for, and objectives of, the Systematic Review clearly stated?

Yes

Is the statistical analysis and its interpretation appropriate?

Not applicable

Are sufficient details of the methods and analysis provided to allow replication by others?

Yes

Are the conclusions drawn adequately supported by the results presented in the review?

Yes

Reviewer Expertise:

Neuroscience, Cardiology, Electrophysiology and Pulmonology

I confirm that I have read this submission and believe that I have an appropriate level of expertise to confirm that it is of an acceptable scientific standard.

# Peer review: Reviewer response for version 1

**DOI:** 10.5256/f1000research.124651.r150876

**Published:** 2022-10-10

**Authors:** Syed-Rehan Hussain, Israel Cotzomi-Ortega

**Affiliations:** 1 Division of Allergy & Immunology, Center for Clinical and Translational, Abigail Wexner Research Institute, Nationwide Children's Hospital, Columbus, OH, USA; 2 Abigail Wexner Research Institute, Nationwide Children's Hospital, Columbus, Ohio, USA

This systematic review by the authors aimed to find a pathophysiological link between early atherosclerosis and rheumatoid arthritis based upon current and previous 10 years of peer reviewed studies. They have three clearly stated objectives i) what is the prevalence of premature atherosclerosis in RA. ii) The role of cellular infiltrates, NETosis and cytokine dysfunction etc. in disease. iii) Early predictive markers for atherosclerosis in RA.

 Although it is a fairly interesting review, I have some queries and concerns about the limited number of supporting articles and some conclusions drawn from these studies.

1. “High prevalence of atherosclerosis” on page1 is a subjective term please include percentage/quantifiable value in RA patient.
2. Page 3 what proinflammatory genes are normally upregulated give examples.
3. Give full names for acronym like “CV”.
4. The authors mention in the abstract and conclusion that NETs are playing a role in RA-mediated atherosclerosis. However, they are not supporting this claim by multiple studies. Kindly explain why one study is enough to find the association between NETs formation, RA and atherosclerosis when in the text on page 17 the authors state that the current data is not sufficient to draw a definitive conclusion.
5. Kindly explain why in the introduction osteocalcin (OCN) and osteoprotegerin (OPG) are referred as early biomarkers, however on page 18 they show OCN and OPG association after 11 years of disease progression not at an early stage.
6. Regarding only 73 publications being selected out of over 21000. It seems to be very stringent as in 2021 alone there may be 35-40 publication that may fall in the area of “Rheumatoid arthritis and atherosclerosis”. Kindly elaborate why some relevant publications we not cited particularly where the claims for NETosis and early markers were made.
7. The presence of citrullinated proteins is not only for NETs. Rather it would be interesting to discuss other activities of neutrophil that causes endothelial damage, such as oxidative burst in the context of atherosclerosis.

Are the rationale for, and objectives of, the Systematic Review clearly stated?

Yes

Is the statistical analysis and its interpretation appropriate?

Not applicable

Are sufficient details of the methods and analysis provided to allow replication by others?

Partly

Are the conclusions drawn adequately supported by the results presented in the review?

Partly

Reviewer Expertise:

My area of research is in immunology and viral infections. With some experience in autoimmune disease and gene therapy. My co-reviewer Dr Israel Cotzomi-Ortega  has experience in the role of neutrophils  and infections in kidney disease

We confirm that we have read this submission and believe that we have an appropriate level of expertise to confirm that it is of an acceptable scientific standard, however we have significant reservations, as outlined above.

# Peer review: Reviewer response for version 1

**DOI:** 10.5256/f1000research.124651.r150875

**Published:** 2022-09-30

**Authors:** Srinivasa Rao Sirasanagandla

**Affiliations:** 1 Department of Human & Clinical Anatomy, College of Medicine & Health Sciences, Sultan Qaboos University, Muscat, Oman

I reviewed the article titled "Accelerated atherosclerosis in rheumatoid arthritis: a systematic review." The aim of this study is to assess the prevalence of premature atherosclerosis in RA patients and elucidate the role that proinflammatory cytokines, neutrophil extracellular traps, RA-related autoantibodies, and endothelial dysfunction play in the pathophysiology of RA-mediated atherosclerosis.

 The paper presented is interesting, and overall, it is well written. However, I have the following comments:

- In the first paragraph of the methods section and in the inclusion criteria, it is mentioned that "the search duration was set from 2011–2022. However, in the Prisma table/chart, it mentions the years 2012–2022. Authors should clarify and correct the actual years of inclusion.
- For the results section, it would be better to add all studies with similar study designs in sequence. For example, all prospective studies in sequence. This will be clearer to the readers. Add a letter "n" in the heading of study design and population column.
- In the discussion, please add a paragraph on current therapies to treat RA. In addition, authors can discuss research gaps and add future recommendations before concluding their findings. 

Are the rationale for, and objectives of, the Systematic Review clearly stated?

Yes

Is the statistical analysis and its interpretation appropriate?

Not applicable

Are sufficient details of the methods and analysis provided to allow replication by others?

Yes

Are the conclusions drawn adequately supported by the results presented in the review?

Yes

Reviewer Expertise:

Natural products, osteoporosis, cardiovascular research and Bisphenol A associated toxicity and morphological variations.

I confirm that I have read this submission and believe that I have an appropriate level of expertise to confirm that it is of an acceptable scientific standard.

# Response

**Published:** 2022-09-30

**Authors:** Vasavi Gorantla

**Affiliations:** St. George's University School of Medicine , Grenada

Thank you Doctor for your review. We will incorporate all the suggestions made.

 Regards,

 Gorantla
